# The role of excess charge mitigation in electromagnetic hygiene: An integrative review

**DOI:** 10.1016/j.bj.2024.100801

**Published:** 2024-11-03

**Authors:** Isaac A. Jamieson, J. Nigel B. Bell, Paul Holdstock

**Affiliations:** a Thammasat University Research Unit in Resilient Innovation, Faculty of Architecture and Planning, Thammasat University, Rangsit Center, Pathumthani, Thailand; b Centre for Environmental Policy, Faculty of Natural Sciences, Imperial College London, London, United Kingdom; c Holdstock Technical Services, Manchester, United Kingdom

**Keywords:** Electromagnetic hygiene, Infection prevention and control, Particulate matter, ESD mitigation, Productivity, Humidity

## Abstract

The electromagnetic characteristics of many environments have changed significantly in recent decades. This is in large part due to the increased presence of equipment that emits electromagnetic radiation and materials that may often readily gain excess charge. The presence of excess charge can often increase the risk of infection from pathogens and the likelihood of individuals experiencing compromised performance, respiratory problems, and other adverse health issues from increased uptake of particulate matter. It is proposed that adopting improved electromagnetic hygiene measures, including optimized humidity levels, to reduce the presence of inappropriate levels of electric charge can help reduce the likelihood of ill health, infection, and poor performance arising from contaminant inhalation and deposition, plus reduce the likelihood of medical devices and other electronic devices getting damaged and/or having their data compromised. It is suggested that such measures should be more widely adopted within clinical practice guidelines and water, sanitation, and hygiene programs.

## Highlights

• Electric fields can increase localized deposition of pathogens.
• High charge of either polarity increases contaminant deposition.
• 40–60% relative humidity reduces likelihood of infection.
• Proper specification of materials reduces infection risks.
• Electromagnetic hygiene can reduce risk of infection.

## 1 Summary

Electromagnetic hygiene protocols are measures and practices considered advantageous to maintaining and enhancing wellbeing and performance whilst reducing the likelihood of ill-health by helping optimize the bio-friendliness of electromagnetic environments [1].

Excess charge is often encountered in the present-day world and can increase the probability of ill-health though increasing local surface contamination and retention of inhaled contaminants [2]. The economic burden placed on healthcare systems due to healthcare-associated infections (HAI) alone is substantial [3]. They are the most common issue affecting patient safety [4] and can cost ≤6% of hospital budgets [5]. Furthermore, unless infections from antimicrobial resistant pathogens are reduced, they could cost the world economy around US$100 trillion in terms of lost production from 2015 to 2050 and be responsible for around 10 million mortalities annually by 2050 [6]. Excess charge can also contribute to illness and economic burdens created through inhaling particulate matter (PM) by increasing its deposition in the airways [7]. Reduced cognitive functioning can additionally occur through PM uptake [8], as can increased risk of early death from exposure to PM, even at levels within legal limits [9]. Furthermore, adverse patient health outcomes can, in part, arise from electrostatic discharge (ESD) events [10] that can compromise staff efficiency and effective use of medical equipment, including mobile devices used for medical purposes.

This review discusses how excess charge can contribute to adverse outcomes and suggests how such risks can be addressed through evidence-based electromagnetic hygiene charge strategies that can be incorporated into expanded clinical practice guidelines (CPG) and water, sanitation, and hygiene (WASH) programs.

## 2 Triboelectric charging and humidity

Triboelectric charging can generate high charge and occurs when materials in contact with each other are separated. When rubbing/frictional movement is also involved, the degree of charging is greater than through separation alone.

Particularly high charging often occurs when materials at opposite ends of a triboelectric series are involved [[11], [12], [13], [14]] [Table 1a], and in low relative humidity (RH) due to low moisture levels in the air and on surfaces reducing the ability of the environment to dissipate or neutralize charge. As an example, walking across a vinyl floor at low RH generated an electrostatic potential of 12 kV compared to only 0.25 kV at higher RH [15] [Table 1b]. Low RH conditions can often arise during wintertime and when heating and HVAC are used [16] unless proactive measures are taken [2].

**Table 1:** Triboelectric charging.

[Table 1a]: Triboelectric series (partial listing) [[11], [12], [13], [14]] Materials in descending order from positive to negative ends of series, i.e., from those most apt to give up electrons to those least likely to surrender them	[Table 1b]: Human body voltages from triboelectric charging at different RH [15]		
	Activity	Human body voltage (kV)	
		10–20% RH	65–90% RH
Positive: Dry air → Polyurethane foam → Hair → Nylon, dry human skin → Plexiglas → Asbestos → Leather → Silicon wax → Cellulose acetate → Silicone → glass → Polyformaldehyde → Human hair → Ethyl cellulose → Polyamide (PA) → Nylon → Wool → Cat Fur → Silk → Aluminium → paper → cotton →	Walking across a carpet	35	1.5
	Walking across a vinyl floor	12	0.25
	Working at a bench	6	0.1
Neutral: Steel			
Negative: Wood → Acrylic → polystyrene → Rubber → Resins (natural & man-made) → Hard rubber → Nickel, Copper → Brass, Silver → Gold, Platinum → acetate, Rayon → Synthetic rubber → Dacron® (polyester fibre) → Orlon® (synthetic fibre used in fabrics, imitation Fur and carpets) → Styrene (styrofoam) → Saran Wrap (cling film) → Polyurethane → polystyrene → natural Rubber → polyethylene terephthalate glycol (PETG)] → polypropylene (PP) → Polyvinyl Chloride (PVC) → Silicon → Polytetrafloroethylene (Teflon) → Silicone rubber → Ebonite	Opening a vinyl envelope for work instructions	7	0.6
	Polyethylene bag picked up from bench	20	1.2
	Rising from a chair padded with polyurethane foam	18	1.5

Wherever possible, RH should be between 40 and 60% RH [1,15,[17], [18], [19], [20]]. Taking measures to achieve this can help reduce the likelihood of infection and PM uptake whilst helping increase worker performance and reducing the possibility of electrical equipment and data being compromised. A strong case can be made for careful selection of materials plus advocating 40–60% RH levels to reduce charge generation [1,15,20] and contaminant deposition [7,21,22]. Furthermore, adiabatic humidification systems can be used to optimize humidity in healthcare facilities [23] and deliver energy savings over traditional isothermal/steam humidification used for such purposes [24].

## 3 Electrical charge held by microbes and effects of charge on microbe deposition

Many activities can generate charge at levels that increase pathogen deposition and the likelihood of infection. Microbes themselves can hold very high net-charges of either polarity. Mainelis et al. [25] investigated the polarity and degree of electric charge carried by airborne bacteria and non-biological particles between 0.65 and 0.8 μm in size. A wide elementary charge range was observed, with higher degrees of charge of either polarity being obtainable for biological particles compared to their non-biological counterparts; sometimes as high as 13,000 or more elementary charges per bacterium [Fig. 1a].

**Fig. 1a:**
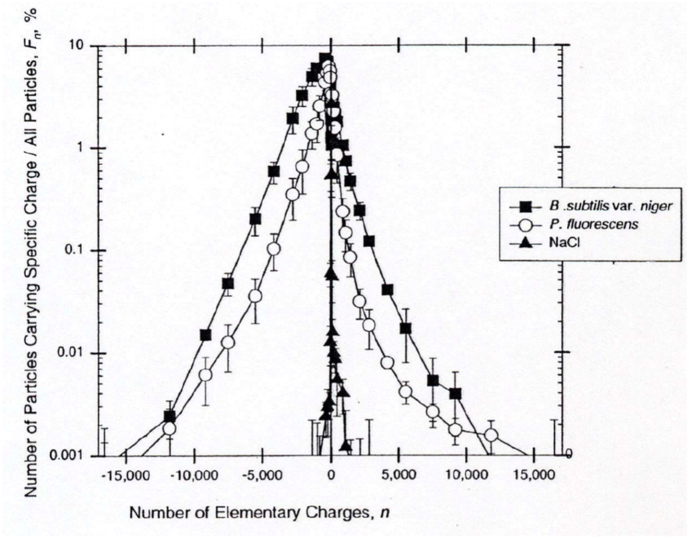
Elementary charge distribution range of airborne bacteria and NaCl when aerosolized [25].

Research by Allen et al. [21,22] indicates how the charge held by a surface can influence the localised deposition of microbes [Fig. 1b]. Both negative and positive potentials increased deposition, which became more pronounced as the charge increased. The charge held by the particles themselves will have also influenced their deposition rates and velocities.

**Fig. 1B:**
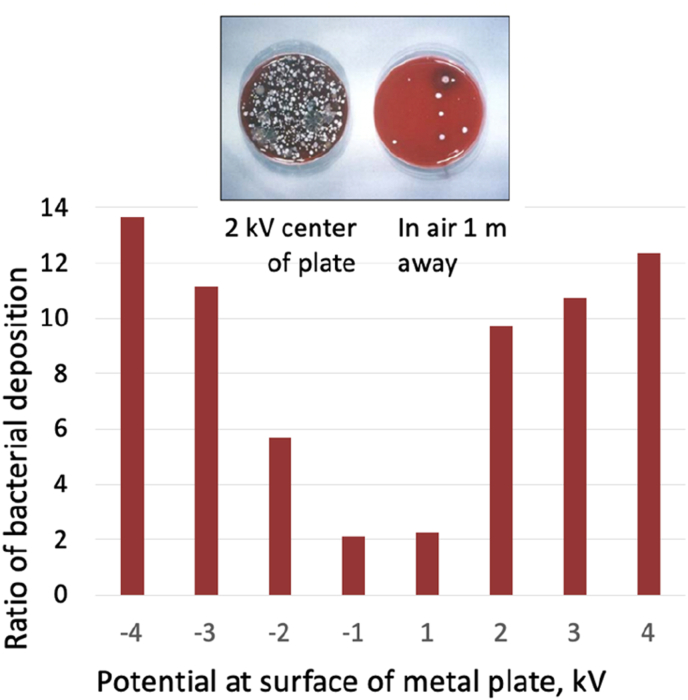
Bacterial deposition on surfaces charged to different potentials. Adapted from Refs. [21,22].

Meschke et al. [26] also observed bacterial deposition rising as surface electrostatic potentials increased and found that surface electrostatic potentials of +5 kV deposition were over twice as high as through gravitational sedimentation alone. Furthermore, deposition rates can influence microbe concentration levels and survival times. Neely [27] observed that at 10^2^ microorganisms per swatch, bacterial survival times ranged from <1 hr up to 8 days, whilst at 10^4^-10^5^ bacteria per swatch, it was 2 hr to >60 days. Electrostatic surface potential is also a key factor in virus transmission [28,29] and fungi deposition [30].

## 4 Particulate matter

Particulate matter is the term given to a mixture of liquid droplets and solid particles present in the air [31]. PM_2.5_ are particles with aerodynamic diameters ≤2.5 μm (μm) in size and PM_10_ are airborne particles of ≤10 μm. Increased PM uptake is associated with increased risk of respiratory symptoms, hospital admissions, cardiovascular and respiratory morbidity, and mortality from lung cancer and cardiovascular and respiratory diseases [[32], [33], [34]]. It is additionally associated with an increased risk of diabetes mellitus [35], chronic kidney disease [36], and pneumonia [37]. In 2019, the global health cost of morbidity and mortality resulting from exposure to PM_2.5_ was US$8.1 trillion [38]. Increased exposures are also associated with reduced mental functioning [8], increased risk of Alzheimer's disease and related dementias, Parkinson's disease [39], depressive symptoms [40], and early death [9].

Excess charge can increase such risks. As noted by Hinds [41], most airborne PM carries electric charge, and some can possess very high charge that can result in their electrostatic force being “thousands of times greater than the force of gravity.” They can often be removed from the air through electrostatic precipitation. Additionally, most airborne particles found indoors are <1 μm [42]; a size range for which electric fields can act as major transport and removal mechanisms [43]. 1 μm particles typically carry between 1 and 10 charges, though inductive charging adjacent highly charged objects can increase this to almost 1000 [44]. The deposition of PM_2.5_ and PM_10_ in human airways can be substantially increased when they possess high charge [7].

## 5 Excess charge in healthcare environments

High levels of excess charge are often experienced in healthcare environments. Such issues are increasing and urgently need addressing [45]. Healthcare personnel can frequently gain body voltages >20 kV whilst undertaking routine activities [46]. Often individuals receive induced electric charge on their bodies when next to sources of raised electric fields, such as inappropriately designed electrical items and/or electrostatically charged materials, even if grounded to reduce excess voltage [1]. They can additionally become charged through triboelectric, or frictional, charging of materials, clothing, and/or footwear [15] [Table 1b]. Objects can also acquire charge induced by electric fields and/or become triboelectrically charged, which can increase localized deposition of airborne contaminants [1,[20], [21], [22]]. Luckily, antistatic treatment of insulating materials can create statistically significant reductions in the airborne contamination they receive [47].

## 6 Clinical activities and excess charge

### 6.1 Personal protective equipment (PPE) and excess charge generation

Inappropriate choice and combination of personal protective equipment (PPE) can lead to the generation of raised levels of charge and associated voltage plus increased infection risk.

Aprons: When nurses wear highly charged aprons, an equal and opposite charge to that of the apron can be induced on individuals they treat which can attract airborne pathogens towards them. The materials specified can significantly influence the degree of charge generated. As an example, Allen & Henshaw [48] document mean electrical potentials at apron pull-off at dispenser and during its wear of −1.16 (−0.38 to −2.92) kV and +0.036 (−0.175 to +0.205) kV for an antistatic apron, compared to −4.49 (−1.43 to −9.62) kV and −0.278 (−0.107 to −0.565) kV, respectively, for a standard disposable white plastic apron. That work revealed a 38% reduction in bacteria attracted to the antistatic apron type compared to the disposable white plastic aprons being tested.

Gloves, gown, and uniform: The highest potentials obtained by Badran et al. [49] from the contact and separation of a doctor's uniform and a medical gown were +750 V and −900 V, respectively. Sliding a glove across the gown's surface caused excess charging of up to +2100 V and −2600 V, respectively. Additionally, some types of gloves generate significantly higher voltage than others [50]. As an example, one type of latex glove generated a triboelectric potential of 701 V during testing compared to 25 V for a white nitrile glove and 17 V for a white cloth glove. Moreover, using static dissipative gloves with anti-static properties appears preferable to latex or vinyl gloves when wishing to avoid raised charge and slow static decay times that increase the likelihood of pathogen deposition.

#### 6.1.1 Eye protective devices and safety screens

These can generate high levels of charge if incorrectly specified and/or used. High charge can attract increased numbers of charged and charge-neutral airborne contaminants towards eyes, faces, and personal breathing zones, increasing the risk of inhalation and infection.

Face shields: Electrostatic potentials of between −2000 and −2700 V have been recorded on the outer surface of a polypropylene (PP) face shield. When the test subject wore a PP face mask in addition to the face shield, potentials of up to −4900 V were recorded [51].

Goggles: Polymethyl methacrylate (PMMA) goggles rubbed with PP, cotton, or paper reached a potential of +11,000 V. They also gained high levels of charge after being rubbed by gloves, especially nitrile gloves resulting in potentials of up to +17,000 V being generated. Goggles rubbed by bare fingers created a negative potential of −1350 V [11].

Spectacles: These are often rubbed to reduce misting when wearing masks. One lens type tested registered an electrostatic potential of +5000 V after being rubbed, whilst another generated around +1000 V. The charges of both decreased with time [51].

Safety screens: Protective safety screens are often made of materials that act as electrical insulators and gain charge readily. Research investigating the deposition of 3.5–9.0 μm particles onto vertical surfaces under the influences of electrostatic forces revealed deposition velocities were significantly higher for acetate sheet than plain glass. Anti-static treatments reduced deposition onto acetate sheet by 93% and the surface of glass by 83% [52].

#### 6.1.2 Atmospheric and airborne microplastics and nanoplastics (aMP)

Plastics, including those used for PPE, release aMP, a recognised contaminant of concern [53], through general wear and tear. aMP can carry far higher charges than many ‘natural’ types of particles [54], and act as vectors of primary air pollutants [55]. They are consistently reported in areas where high levels of human activity are undertaken. As an example, Field et al. [53] recorded a mean of 1924 ±3105 aMP/m^2^/day in an operating theatre compared to 541 ±969 aMP/m^2^/day in an adjacent anaesthetic room. The most abundant polymer types identified for aMP can all gain and retain high charge. Furthermore, aMP has been detected in human lung tissue [56], and it has been suggested that: “By adhering to the surface of microplastics, microorganisms may be directly transported to the human lung, circumventing defense mechanisms and possibly resulting in infection, especially in debilitated areas already suffering from particle toxicity” [57]. Additional concern arises from the infection risk this type of exposure may cause during surgery [53], and that excess charge will increase deposition.

## 7 Charge generation by moveable items used in hospitals

In research by Viheriäkoski et al. [46], undertaken at ≈27% RH, electrostatic potentials of ≤30 kV were recorded on ungrounded moveable objects such as chairs, delivery carts, overbed tables, patient beds, intravenous stands, and trolleys. The maximum recorded human body potentials recorded moving such items were >20 kV. Measures to help reduce charge build-up include using the right combinations of materials and finishes, having appropriate RH levels, moisturizing the skin, grounding individuals via footwear and flooring, grounding conductive objects, and having either conductive or static dissipative wheels on moveable metal equipment and furniture [1,2,46]. Additional factors for ‘biological grounding’ of individuals are discussed by Jamieson [1].

### 7.1 Excess charge generated at hospital beds

Standard activities, such as changing posture and getting up from chairs and beds, too can generate increased body voltages, which attract airborne contaminants towards individuals. In research by Kohani et al. [58] undertaken at ≤30% RH, peak body voltages >15 kV arose in almost half of the people lying down in hospital bed tests and 38% of tests transferring an individual to a hospital bed using a sliding board. These values exceed the permitted 15 kV test voltage level for protecting against electrostatic discharge (ESD) in the International Electrotechnical Commission's IEC 60601-1-2 standard [59]. Those authors additionally reported that having 20% RH in hospitals instead of 30% RH would increase charging voltage of the body by 27% [58].

**Fig. 2:**
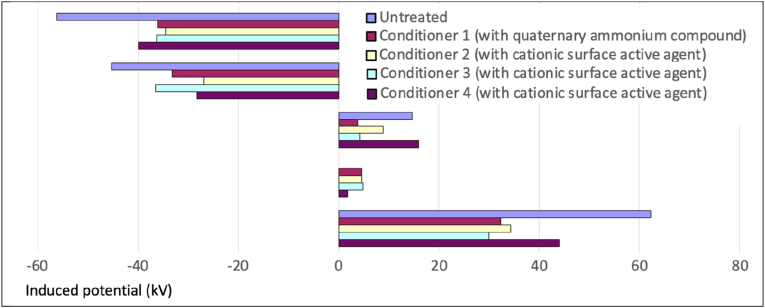
Induced potentials on humans removing hospital bedding (After Holdstock & Wilson [20], with kind permission of the EOS/ESD Association, Inc.).

Bedding exchange can also generate high potentials. Endo et al. [60] showed that whilst the degree of charge cotton bedclothes gained during bedding exchange was strongly dependent on RH levels, the charge polyester bedclothes gained was less dependent on humidity and could still induce human body potentials >10 kV even at 50% RH. Holdstock & Wilson [20] reported whilst the use of topical finishes can reduce charging levels, in worst-case scenarios removing blankets and sheets from hospital bedding when there was an inappropriate combination of materials and low RH (33 ± 2% RH) could generate direct current (DC) body voltages >60 kV [Fig. 2].

It is proposed that in such situations, whilst paying close attention to the kinds of materials and finishes that come into contact with each other, bipolar ionization and optimized humidity levels should also be considered as methods of static control.

## 8 Excess charge generated during walking

Walking activities can significantly increase dust and particle resuspension and generate high electrostatic potentials [61,62]. The level of charge generated depends on a variety of factors, including the triboelectric characteristics of materials and RH (see [Table 1]), and whether people lift their feet or shuffle (which further increases charge generation) when they walk [2]. Additionally, microbes, dust, and particles emitted from the surface of objects through movement and triboelectric (frictional) charging can be repelled by those objects when they share the same polarity and become attracted to objects of the opposite polarity. Electrostatic shocks from carpeting are far more likely at <40% RH [63].

### 8.1 Resuspension of floor dust containing microbes

Bacteria: Resuspended floor dust can exhibit significantly greater concentrations of bacteria relative to indoor air, outdoor air, and ventilation duct supply air. In research by Hospodsky et al. [64], the median bacterial mass percentages of resuspended floor dust for PM were almost one order of magnitude larger than for indoor and outdoor airborne particles (>2.2% compared to <0.3%).

Fungi: Measurements taken at floor level, 1 m and 1.5 m heights revealed bioaerosol concentrations highest at 1 m level after walking activities. Flooring type strongly influenced resuspension rates, with viable airborne concentrations of Penicillium chrysogenum spores being significantly greater after walking activities on cut pile carpeting compared to walking on loop pile carpeting or vinyl tile flooring [65].

Viruses: Research investigating the vertical concentration gradient of influenza viruses in floor dust resuspended through walking indicated ≤40% greater concentrations of resuspended viruses at 1 m compared to 2 m above the floor, contingent on particle size [61]. This appears due in part to the high degree of turbulence 0.75–1 m above flooring caused by swinging of arms whilst walking. Additionally, low RH significantly increased airborne viral concentrations compared to higher RH. Increased concentrations of resuspended viruses at 1 m above floors will further increase the likelihood of their deposition onto the hands of those walking, especially if individuals become highly charged [15] and more likely to attract airborne contaminants. Damp cleaning of hard surfaces can help reduce contaminant resuspension [2].

## 9 Electrostatic discharge events

Personnel ESD events (electrostatic shocks) are painful to individuals and, in some situations, trigger involuntary movements that could cause accidents. Viheriäkoski et al. [46] undertook a preliminary survey on ESD events experienced by nurses during a dry winter period. Only 8% considered such events insignificant, 15% thought them a light nuisance, 23% a nuisance, 23% a strong nuisance, and 31% considered them unbearable. Regarding how often they experienced these at that time, 23% received them a few times a month at work, 23% a few times a week, 31% a few times per day, and 23% received them several times daily. Some said they affected their wellbeing and work practices. With regard to what they were adjacent to when experiencing these events, 70% had been next to patient beds, 46% next to a metal part of an elevator, 46% next to other metal objects, 38% next to a colleague, 38% next to a patient, 31% next to a door handle, and 8% next to medical equipment.

ESD events can also increase the risk of electronic devices failing, malfunctioning, or having their data corrupted. They can additionally increase the likelihood of medical staff avoiding using particular devices to avoid receiving painful shocks. The analysis by Kohani & Pecht [10] of ESD malfunctions of medical devices unveiled 5 reports of patient deaths and 46 injuries that may have arisen at least in part due to ESD events. For class III medical devices, ESD malfunctions were noted in all death reports and 20 injury reports. They also found 90 incidences where re-implantation of cochlear implants or neurostimulators was necessary due to such events. Additionally, malfunctions of medical devices resulting from ESD events were almost six times greater in cold weather when humidity is low.

The IEC recommends that the surface or volume resistance of electrostatic dissipative materials should be ≥ 1 × 10^4^ Ω and <1 × 10^11^ Ω to help reduce the generation of electrostatic charge and dissipate charge slowly enough to prevent ESD [66]. It is important to note that many materials used to help address ESD risks typically do not work well below 20–30% RH and that some can act as electrical insulators in such conditions [67]. Such findings further emphasize the need for correct specification of both materials and humidity levels in electromagnetic hygiene initiatives.

## 10 Excess charge and respiratory health

The prevalence of asthma is increasing and costs the US economy alone > US$80 billion annually in terms of healthcare costs, lost schooldays and workdays, and early mortalities [68]. In addition to PM_2.5_ exposure being associated with asthma prevalence [69], there is a strong correlation between the presence of raised excess charge and incidents of asthma, with it being indicated that antistatic treatment of the environment and wearing clothing that generates little charge can greatly reduce the likelihood of asthma attacks [[70], [71], [72]]. We suggest this may be due to there being reduced concentrations of charged airborne contaminants in individuals’ personal breathing zones.

### 10.1 Cleanliness of air

Enhanced indoor air quality can significantly improve individuals’ cognitive response with regard to crisis response, information usage, and decision-making abilities [73]. lmproved air renewal rates and air quality can help reduce the likelihood of infection [74]. The benefits of taking approaches to achieve these were championed by Florence Nightingale [75] over 160 years ago, and the advantages of adopting health-focused ventilation rates remain apparent to this day [76,77].

Air ion levels can provide a good indication of the cleanliness of air, with bipolar concentrations of 600 negative small air ions/cm^3^ (NSAI/cm^3^) and 400 positive small air ions/cm^3^ (PSAI/cm^3^) being recommended as absolute minimums for electromagnetic hygiene purposes. The optimum levels are 3000–5000 NSAI/cm^3^ and 1500–3000 PSAI/cm^3^ [78]. Interestingly, mice infected with influenza experiencing almost similar exposures survived longer and had lower mortality rates than those exposed to lower concentrations [79].

**Fig. 3:**
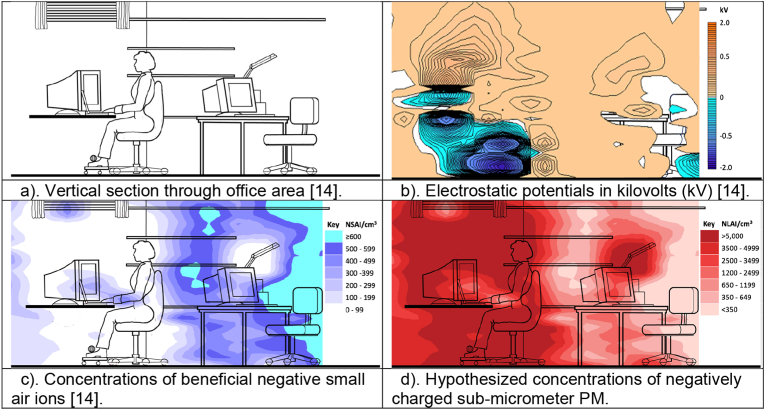
Cross-section through radiographers' suite in Bergen, Norway.

Raised concentrations of charged and charge-neutralized submicron particles that can cause health issues are often present in areas where raised electric fields and low concentrations of small air ions co-exist [14,80] even when there is good ventilation [14]. [Fig. 3a and b, c] show a radiographer's office and the electrostatic potentials and concentrations of NSAI measured in it [14]. [Fig. 3d] shows the hypothesized concentrations of negatively charged sub-micrometer PM (negative large air ions (NLAI)) that the lead author believes would have arisen and suggests individuals sitting at the computer workstation would have experienced increased exposure to airborne contaminants, contaminant deposition and retention of inhaled PM and airborne microorganisms compared to if they had been occupying a low field microenvironment.

### 10.2 Influence of charge on particle deposition in human airways

Electrostatic deposition is one of the direct mechanisms of deposition of respiratory aerosols in airways [41]. Increased deposition of submicrometer and micrometer particles, including human skin flakes, occurs when particles carry greater charge. Xi et al. [7] report that for particles between 0.4 and 10 μm nasal deposition for particles with a high level of charge was an order of magnitude greater than for neutral particles of similar size.

### 10.3 Survival times of pathogens on human skin

The survival times of pathogens on human skin vary. As an example, Hirose et al. [81] report that whilst influenza A virus can survive around 1.8 h on skin, the survival period of SARS-CoV-2 is significantly longer at around 9 h.

## 11 Particle deposition onto human skin

### 11.1 Excess charge and particle deposition onto the human face

Human skin can gain high potentials, especially when RH is low [82]. The influence raised electric field strengths can have on particle deposition rates and velocities is quite pronounced. Charged particles can rapidly attain their terminal velocities in uniform electric fields. As an example, a 0.01 μm particle with a single charge that would travel at 0.0021 cm per second (cm/s) in a 0.010 kV per meter (kV/m) field would travel at a velocity of 2.1 cm/s in a 10 kV/m electric field. Even higher mean induced field-strengths of 95–100 kV/m can arise on individuals' faces 0.40 m from a +2 kV DC source. This is because increased field strengths can arise at face areas that act as ‘point emitters’ and concentrate charge [83].

Wedberg [84,85] demonstrated that the deposition of airborne contaminants onto human faces can be significantly influenced by the degree to which an individual is charged. He reported deposition of PM (>0.07 μm minimum resolved diameter) at rates of ≈100 particles/mm^2^/hr at 0 kV conditions, ≈1000 particles/mm^2^/hr under moderately high body potentials of ±5–6 kV, and >10,000 particles/mm^2^/hr under still higher potentials.

### 11.2 Deposition velocities of particles onto hands

Andersson et al. [86] assessed deposition velocities of airborne particles onto hands 20 cm or 70 cm from a surface potential of 11–13 kV. The deposition velocity of 0.7 μm particles was significantly greater onto hands 20 cm from the source rather than 70 cm from it. Additionally, Becker et al. [87] investigated the extent to which electrostatic fields could act as a mechanism for bacterial transfer to patients from gloved fingers that had been close to a highly charged object. They observed bacterial growth on >93% of cultures taken at distances of ≤4 cm from the source, whilst no growth was shown for samples taken at 8 cm from the source or on controls used. They additionally reported the number of bacterial colonies cultured from the glove tips was inversely proportional to the distance glove tips had been from the field source. The types of gloves worn will also influence deposition rates, with those that gain higher charge [50] likely to experience greater deposition.

## 12 Skin flakes

### 12.1 Skin scales and micro-organism shedding

Skin flakes can become highly charged [2]. On average, around a million skin flakes of between <1 and 50 μm in size are shed by the human body every minute [88]. They form the greatest source of PM within individuals’ personal breathing zones. Typically, between 6000 and 50,000 skin flakes of 5–50 μm size are inhaled per litre of nasally-inhaled air [89].

Infectious bacteria, viruses, and fungi on the surfaces of skin cells can become airborne on shed skin scales [90]. Around 5–10% of all shed skin scales can harbor bacteria [91]. Contaminated skin flakes can themselves be a source of microbial infection during surgical procedures, plus cause other nosocomial infections [92] and infections arising in everyday situations. Their contaminant loading and chances of being retained when inhaled can be higher in situations where increased charge is present.

Reducing the release of contaminated skin flakes lessens the likelihood of infection through their deposition in the airways, on open wounds, or on nearby surfaces. Moisturizing skin is an inexpensive way to achieve this and can reduce the dispersal of pathogens on skin flakes to a level at least as good as that achieved by protective clothing [93]. It can also reduce frictional charging, thereby reducing body potentials and the number of airborne contaminants attracted towards individuals, help reduce the level of charge released skin flakes hold, and aid the effectiveness of grounding measures to reduce body potentials [1,2].

## 13 Effects of humidity on biological contaminants and likelihood of infection

Sterling et al. [19] investigated the indirect effects of different RH levels on health, including bacteria, virus and fungi viability [Fig. 4a]. That work indicated an optimum zone of between 40 and 60% RH where possible adverse effects can be most reduced and that, where possible, very low and very high humidity levels should be avoided. Research shows lung lesions and mortalities in animals exposed to aerosolized influenza minimized at 40–60% RH compared to higher and lower humidities [63]. The association between RH and the likelihood of infection is further demonstrated by Taylor & Hugentobler [18], who observed a marked increase in patient HAI in patient rooms with <40% RH [Fig. 4b]. In 2007, the estimated annual direct medical costs of HAI in the US alone were ≤US$45 billion [94], with a further US$12.4 billion in costs to society from lost productivity and early deaths [95], highlighting the need to take such findings seriously.

**Fig. 4a:**
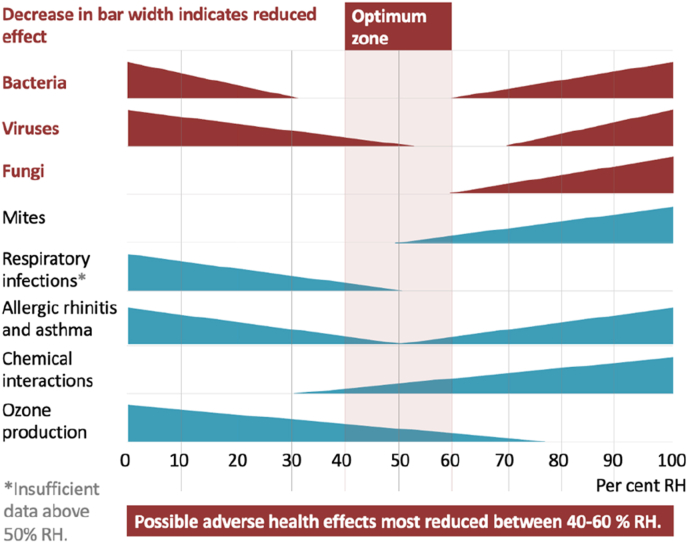
Optimum ranges of relative humidity for health [19].

**Fig. 4b:**
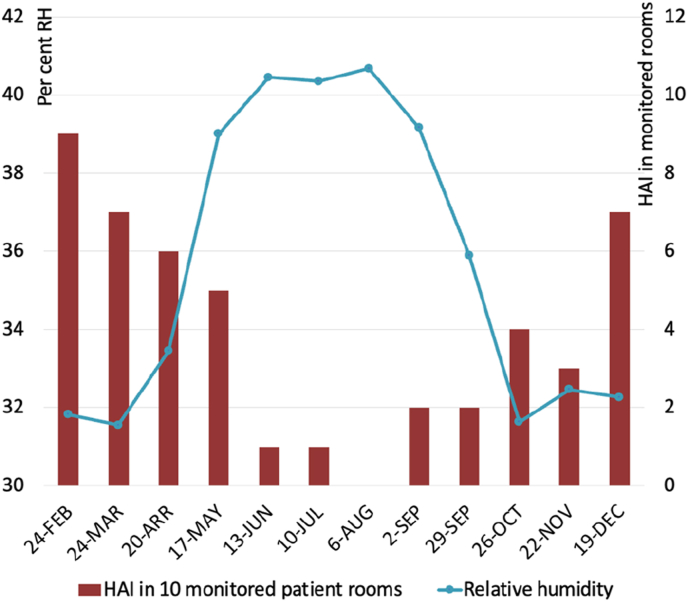
Correlation between average RH levels and patient HAIs [18]. Used with permission.

### 13.1 Effects of humidity levels on mucociliary clearance rate, fatigue, and cognitive performance

Low RH levels reduce the mucociliary clearance rate of upper airways (a factor increasing the likelihood of infection). They also reduce the stability of precorneal tear film of the eyes (which increases likelihood of dry eyes and visual fatigue). Having 40–60% RH can help address such risks [96].

Reductions in cognitive performance can also arise under inappropriate humidity levels. Liu et al. [97] observed that, compared to individuals in environments at 20% RH, those in areas at 40% RH exhibited improved learning performance: 61.1% lower degree of distraction, 1.44% improvement in reading accuracy, 12.2% faster reading speed, and 23.3% lower degree of fatigue. This is particularly important to consider in healthcare environments, as 20% RH is presently permitted in some healthcare situations [98] and fatigued workers are more likely to make safety-critical errors. Increased levels of fatigue are additionally associated with reduced satisfaction, plus increased staff turnover and negative patient health outcomes [99].

### 13.2 Insulative materials and excess charge

Even when humidity is optimized, attention should be given to the possible contributory effects of materials as related to charge generation and pathogen deposition. As an example, Allen et al. [48] reported that the number of bacterial colonies incubated from the surfaces of antistatic aprons after pull-off from a dispenser and 10 min wear was 38% lower than for standard plastic nurses’ aprons at ≥57% RH. Furthermore, research by Cozanitis et al. [47] shows coating insulating objects with antistatic solution can lessen their electrical resistance, as a result of which deposition of airborne pathogens is reduced. In particular, excess charge did not accumulate when insulating resistance was <10^9^–10^10^ Ω.

## 14 Conclusion

Excess charge can be a contributory factor to ill health through increasing localized deposition of contaminants. Microenvironments with raised electric fields can also exhibit poorer air quality and greater concentrations of charged PM that have a higher likelihood of deposition in airways than uncharged PM. The presence of excess charge, particularly in conditions of low RH, can also lead to individuals experiencing painful electrostatic shocks and electrical equipment being damaged and/or having data compromised through ESD events. Furthermore, low RH can significantly reduce biological and cognitive performance [17,96,97].

Fortuitously there are measures such as optimizing humidity levels, specifying the right types and combinations of materials and topical finishes to reduce charge generation, use of appropriately designed electrical items, grounding of conductive objects and biological grounding, moisturizing the skin, and improving bipolar SAI levels which can help address such challenges and, it is proposed, allow significant financial savings to be made whilst helping increase wellbeing, staff performance, and reduce risk. International Standards IEC 61340-6-1 (healthcare facilities) [100] and IEC TS 61340-6-2 (public spaces and office areas) [66] provide guidelines and requirements for appropriate control measures and materials.

It is hoped that the information provided in this review will be of benefit to public health researchers, policymakers, healthcare officials, and academic and public health research institutions. It is proposed that electromagnetic hygiene strategies should become more widely adopted within WASH programs and CPG, and that doing so will help healthcare services and others use their resources more effectively. The need for further research and action in this area is strongly indicated.

## Declaration of Competing interest

None.
